# Comparative Proteomic Analysis of an Ethyl Tert-Butyl Ether-Degrading Bacterial Consortium

**DOI:** 10.3390/microorganisms10122331

**Published:** 2022-11-25

**Authors:** Vijayalakshmi Gunasekaran, Núria Canela, Magda Constantí

**Affiliations:** 1 Departament d’Enginyeria Química, Universitat Rovira i Virgili, Av. Països Catalans, 26, 43007 Tarragona, Spain; 2 FA Bio, Harpenden AL5 2JQ, UK; 3 Centre for Omic Sciences (COS), Joint Unit Universitat Rovira i Virgili-EURECAT, Av. Universitat 1, 43204 Reus, Spain

**Keywords:** ETBE, biodegradation, bacterial consortium, metaproteome, 2D-DIGE

## Abstract

A bacterial consortium capable of degrading ethyl *tert*-butyl ether (ETBE) as a sole carbon source was enriched and isolated from gasoline-contaminated water. *Arthrobacter* sp., *Herbaspirillum* sp., *Pseudacidovorax* sp., *Pseudomonas* sp., and *Xanthomonas* sp. were identified as the initial populations with the 16S rDNA analysis. The consortium aerobically degraded 49% of 50 mg/L of ETBE, in 6 days. The ETBE degrading efficiency of the consortium increased to 98% even with the higher concentrations of ETBE (1000 mg/L) in the subsequent subcultures, which accumulated tert-butyl alcohol (TBA). *Xanthomonas* sp. and *Pseudomonas* sp. were identified as the predominant ETBE degrading populations in the final subculture. The metaproteome of the ETBE-grown bacterial consortium was compared with the glucose-grown bacterial consortium, using 2D-DIGE. Proteins related to the ETBE metabolism, stress response, carbon metabolism and chaperones were found to be abundant in the presence of ETBE while proteins related to cell division were less abundant. The metaproteomic study revealed that the ETBE does have an effect on the metabolism of the bacterial consortium. It also enabled us to understand the responses of the complex bacterial consortium to ETBE, thus revealing interesting facts about the ETBE degrading bacterial community.

## 1. Introduction

Fuel oxygenates are oxygen-rich compounds that are added to reformulated gasoline to enhance the octane number, and increase the combustibility of the gasoline, thus reducing toxic emissions. When ether fuel oxygenates, such as methyl tert-butyl ether (MTBE) and ethyl tert-butyl ether (ETBE), were added to gasoline, the toxic lead was replaced and the emissions of carbon monoxide, unburned hydrocarbons, polycyclic aromatics, oxides of nitrates and particulate carbon decreased [1,2]. MTBE was soon phased out in US, following severe groundwater contamination [3,4] and was replaced by ethanol in US [5] and ETBE in Europe [6,7,8]. ETBE is widely used in Europe and Japan, but the prevalence of ETBE contamination is minimally documented. According to Balseiro-Romero et al. [9], the topsoil from a fuel distribution station in Spain, was contaminated with 1419 μg/kg of ETBE. Another study reported the ETBE distribution in a leaking storage tank at a gas station in France, in which the underground water was contaminated with 300 mg/L of ETBE [10]. The global consumption of ETBE in Europe and Japan in 2011 was about three million metric tons, as reported by Digabel et al. [11]. This increased usage of ETBE has been attributed to the fact that it is produced from renewable bio-based ethanol [12,13] which leads to lower CO_2_ emissions than MTBE [14]. However, ETBE is a water-soluble compound and its continued usage can pose a severe threat as a groundwater contaminant. In addition, ETBE has lower threshold limits (1 µg/L for odour and 2 µg/L for taste) than MTBE (7 µg/L for odour and 15 µg/L for taste), according to Dutch standards [13]. There are other risks associated with the use of ETBE-blended gasoline: for example, it can affect the groundwater quality due to the underground tank leakages, and it can be a health risk to the employees who are exposed to ETBE in manufacturing plants. ETBE and its metabolite, tert-butyl alcohol (TBA), have been reported to have neurotoxicity effects [15], hepatotumorigenicity [16], kidney and liver effects [17] and reproductive toxicity [18] at higher concentrations in animal studies. It is evident that ETBE could become a potential threat to human health, due to its extensive use and, therefore, needs to be eliminated immediately.

Of the various methods used to remediate a chemically contaminated site, the microbial bioremediation is considered to be one of the most significant and cost effective [19]. ETBE has been degraded using isolated strains, such as *Rhodococcus ruber* IFP2001 [20], *Aquincola tertiaricarbonis* L108 [21], *Rhodococcus wratislaviensis* IFP2016 [22], *Rhodococcus* sp. IFP2042 and *Bradyrhizobium* sp. IFP2049 [11]. Microbial communities often collaborate in metabolizing complex substrates to thrive in a chemically contaminated environment. Therefore, it is highly likely that potential ETBE degraders will be lost when colonies are isolated under a particular culture medium in laboratory conditions. However, it is important to obtain indigenous microbial populations from ETBE-contaminated sites if degradation is to be effective. This study aims to enrich and select a bacterial consortium that can degrade ETBE from a contaminated site and proceeds with a novel metaproteomic analysis of the consortium. The enriched ETBE degrading consortium was analysed for its capacity to degrade other compounds, such as TBA, MTBE and benzene, toluene, and xylene compounds (BTX), which are often found together as co-contaminants [9,21]. This study intends to understand the microbial communities on the whole rather than pure cultures. Various methods, such as metagenomics, metatranscriptomics, metaproteomics and metabolomics are available for studying the complex microbial communities [23]. However, metaproteomics were chosen instead of metatranscriptomics, since the gene and protein levels do not always correlate at any given time, in a particular organism [23]. mRNAs are less stable than proteins [24] and the mRNA expression levels do not necessarily correlate with the translational efficiency [25]. Studying proteins would be more interesting, as proteins are the final effectors of any biological response [24] circumventing the limitations involved in the gene expression and posttranslational modifications. Hence, a metaproteomic approach was used to characterize the ETBE degrading communities, to reveal the underlying mechanisms that govern the ETBE degradation, and to identify important enzymes that can be used in bioremediation. Because of the complexity of the proteins in an unknown bacterial consortium, a proteomic analysis was carried out using two-dimensional polyacrylamide gel electrophoresis (2D-PAGE) with difference in-gel electrophoresis (DIGE), in which the proteins are separated, according to the molecular weight and isoelectric point. This enabled us to reduce the complexity of the proteins, improve the resolution and concentrate on the most abundant proteins in the ETBE-induced condition. The downstream proteomic identification was carried out using matrix-assisted laser desorption ionization-tandem time of flight mass spectrometry (MALDI-TOF/TOF-MS). Currently, no single method can provide qualitative and quantitative information about all of the protein components in a complex mixture. Gel-based and gel-free approaches with their respective advantages and disadvantages are complementary and should be used in parallel for a more complete understanding of the protein expression and interactions in a particular physiological condition. This study is an initial approach to the proteome of the bacterial consortium and could be a good starting point for a gel-free proteomic study in the future, which would make the overall analysis richer.

## 2. Materials and Methods

### 2.1. Enrichment of the Bacterial Consortium

A sample of ground water was collected from a gasoline-contaminated water body located in the north-eastern region of Spain. The sample was inoculated at 20% (i.e., 30 mL of sample) in 150 mL of minimal medium (MM) [19] with 50 mg/L of ETBE (from a stock solution prepared in methanol) as the carbon source in a 250 mL conical flask fitted with a screw cap. MM consisted of KH_2_PO_4_—0.225 g/L, K_2_HPO_4_—0.225 g/L, (NH_4_)_2_SO_4_—0.225 g/L, MgSO_4_·7H_2_O—0.050 g/L, CaCl_2_·2H_2_O—0.005 g/L, FeSO_4_·7H_2_O—0.005 g/L along with trace elements (ZnCl_2_—0.1 g/L, MnCl_2_·4H2O—0.03 g/L, H_3_BO_3_—0.3 g/L, CoCl_2_·6H_2_0—0.2 g/L, CuCl_2_·2H_2_O—0.01 g/L, NiCl_2_·6H_2_O—0.02 g/L, NaMoO_4_·2H_2_O—0.03 g/L. The culture was propagated aerobically and incubated at 30 °C at 180 rpm. The first subculture was called consortium B enrichment culture 1 [EB1]). The increase in the biomass was monitored by measuring the optical density (OD) at λ = 600 nm using a Varian Cary 100 spectrophotometer. Following 7 days of incubation, 20% of EB1 was again transferred to 150 mL of fresh MM along with 50 mg/L of ETBE for the second enrichment culture (EB2). Further subcultures were made and incubated in the same conditions and referred to with successive numbers such as EB2, EB3, etc. Each subculture in MM was stored at 4 °C. About eleven subcultures were successively made to obtain a stable enrichment culture. Following three subcultures, ETBE was given as the sole carbon source without the addition of methanol.

### 2.2. Isolation and Identification of the Bacterial Strains

Subculture EB2 was plated onto washed agar plates (as described by Okeke and Frankenberger [26]) amended only with 50 mg/L of ETBE. Then they were incubated at 30 °C until visible colonies appeared. Pure cultures of bacteria were isolated and identified using 16S rDNA identification as described by Gunasekaran et al. [19]. The pure culture of bacteria was isolated and the colonies were inoculated in LB broth and grown for overnight. The genomic DNA was isolated using GenEluteTM bacterial genomic DNA kit (Merck Life Sciences, SLU, Madrid, Spain). The 16S rRNA gene sequences of the bacterial strains were amplified by PCR using the universal primers 616 V (5′-AGA GTT TGA TYM TGG CTC AG-3′) and 699R (5′-RGG GTT GCG CTC GTT-3′). A 1000 bp region of the gene was amplified. The PCR reaction mixtures contained 1X GoTaq Flexibuffer (Promega, Madison, WI, USA), 3 mM MgCl2, 200 μM (each) dNTP, 0.4 μM of each primer, 100 ng of genomic DNA, and 1 unit of GoTaq (Promega, Madison, WI, USA) in a total volume of 50 μL. The optimized PCR conditions were the initial denaturation at 95 °C for 2 min; 25 cycles of denaturing at 95 °C for 30 s, annealing at 55 °C for 25 s; extension at 72 °C for 1 min; and a final extension at 72 °C for 5 min. The PCR fragment was extracted from agarose gel and sequenced with the Sanger sequencing method using the same primers. The 16S rRNA gene sequence results were analyzed using BLAST and the ribosomal database to identify the bacterial species. Similarly, the bacterial colonies from the enriched subculture EB11 were also isolated and identified, as before.

### 2.3. Biodegradation Experiments

**Degradation of ETBE**. Consortium B (EB3) was used to study the degradation of ETBE. About 30 mL of EB3 (20%) was centrifuged and the resulting pellet was washed twice with MM and resuspended in 1 mL of the same. This was inoculated in 150 mL of MM in a 250 mL screw-cap bottle with 50 mg/L of ETBE and incubated in a rotary shaker at 30 °C at 180 rpm. The increase in biomass was monitored by measuring the optical density (OD) at λ = 600 nm. About 10 mL of the sample was collected from each flask at different time points in headspace vials sealed with crimp caps, which were used to estimate the amount of ETBE. Abiotic controls were also used, consisting of a bacteria-free MM medium, amended with ETBE and maintained under the same conditions. Bacterial The consortium B was also tested for its ability to degrade higher concentrations of ETBE. ETBE at concentrations of 50, 100, 500, 1000, 10,000 mg/L was inoculated with the consortium that had undergone eleven transfers (EB11). The flasks were inoculated in duplicate and also had abiotic controls for each ETBE concentration. Then they were incubated in the same conditions, as described above. The ETBE concentration was monitored on the day when visible growth was observed by checking the turbidity of the inoculated flasks (i.e., on day 6 and 16). EB11 was also used to study the degradation of MTBE, TBA and BTX compounds (50 mg/L). The bacterial consortium EB11 (30 mL) was centrifuged and the resulting pellet was washed twice with MM and resuspended in 1 mL of the same medium. This was inoculated in 150 mL of MM in a 250 mL screw-cap bottle with appropriate concentrations of the chemical compounds and incubated in a rotary shaker at 30 °C at 180 rpm, as described above. To quantify ETBE and MTBE, about 10 mL of the sample was collected from each flask at different time points in headspace vials sealed with crimp caps and 1 mL of sample for TBA and BTX.

**Analytical methods.** ETBE and MTBE were quantified by preparing the samples using solid phase micro extraction (SPME). The headspace vials were heated in a water bath for 30 min at 75 °C for ETBE and 55 °C for MTBE and then the gaseous phase was absorbed on an SPME fibre by incubating the fibre into the headspace vials for 30 min at the same temperature. The fibre was then manually injected into the sample inlet of a Hewlett Packard HP6890 capillary gas chromatograph with a DB-624 column (J&W Scientific, Folsom, CA, USA, 30 m × 3320 mm and a 1.8 mm thick film). The FID detector and injector were held at 250 °C. The column was held at 55 °C initially for 3 min and increased to a final temperature of 85 °C, at a rate of 7 °C/min with helium as the carrier gas. Flurobenzene (25 mg/L) was used as an internal standard. The metabolites TBA and hydroxyisobutyric acid (HIBA) during the ETBE degradation assays were identified by direct aqueous injection (DAI) on an Agilent 6890/5793 GC/MSD system with a polar HP-FFAP column (Agilent Technologies, Palo Alto, CA, USA, 30 m × 0.25 mm × 0.25 µm) in splitless mode. The sample was filtered with 0.2 µm filter to remove bacteria and about 1 µL was used as the sample volume. The column was initially set at 50 °C (3.5 min) and increased to 150 °C at a rate of 10 °C/min, the oven temperature was held at 150 °C for 5 min and then increased again to 230 °C at 20 °C/min and held for 20 min. The total run time was of 42.5 min. Helium was used as the carrier gas with a flow rate of 1.5 mL/min.

To quantify the TBA and BTX compounds, the samples were prepared by liquid-liquid extraction using dichloromethane (DCM) as a solvent in a ratio of 2:3. A total of 1 µL of the extracted sample was then injected into the inlet of a Hewlett Packard HP6890 capillary gas chromatograph with FID detector at 250 °C. BTX compounds were separated on an Agilent 190915-433 HP5 5% phenyl methyl siloxane capillary column (30.0 m × 250 µm × 0.25 µm) and TBA on a DB-624 column (J&W Scientific, 30 m × 3320 mm and a film 1.8 mm thick). The injector was held at 250 °C with a split ratio of 30:1. The oven temperature was initially held at 45 °C for 1 min and then increased to a final temperature of 65 °C at a rate of 5 °C/min for 7 min for BTX and TBA. Standards were prepared in triplicate using pure chemicals in DCM.

### 2.4. Proteomic Analysis

**Sample preparation**. The bacterial consortium was inoculated at 20% in 150 mL of MM and incubated at 30 °C at 180 rpm. The bacterial consortium B (EB11) was grown in two different concentrations, 500 (BE500) and 1000 (BE1000) mg/L of ETBE for 16 days (when ETBE was degraded to above 95%) and was used to analyse the protein profile. EB11, propagated in 0.5 g/L of glucose at the same time point, was included as a control. Two independent replicates were used for the control and test samples. The bacterial cells were centrifuged (9000× *g*; 20 min) and the pellets were resuspended and washed three times with phosphate-buffered saline (PBS). The washed pellets were resuspended in 1 mL of the re-suspension buffer (30 mM Tris, 5 mM EDTA, 5 mM MgCl_2_, pH 9) along with 2 mM of the protease inhibitors (Pefabloc SC PLUS, Roche, Germany). They were then subjected to sonication (five cycles of 30 s at 40% amplitude, 50 watts at 30 s intervals) while kept on ice. The lysate was centrifuged at 20,000× *g* for 15 min at 4 °C. For a DNase treatment, the resulting supernatant was then treated with 300 U of Benzonase^®^ Nuclease (Merck Life Sciences, SLU, Madrid, Spain), incubated for 60 min at 4 °C and centrifuged at 20,000× *g* for 15 min. Protein was precipitated by adding 100% of ice-cold TCA to obtain a final concentration of 10% and incubated on ice for 30 min. The samples were centrifuged at 4 °C for 15 min 20,000× *g*. The protein pellet was resuspended in 800 µL of chilled acetone and incubated overnight at −20 °C. The pellet was washed twice with chilled acetone and air dried completely. Finally, the pellet was dissolved in 1 mL of rehydration buffer (RH) [30 mM Tris buffer pH 8.5, 7 M urea, 2 M thiourea, 30 mM DTT and 4% 3-(3-cholamidopropyl) dimethylammoniol-1-propanesulfonate (CHAPS)] at room temperature (30 min with periodical vortexing). The protein samples were then concentrated and cleaned for any inhibitors with the 2-D Clean-Up kit (GE healthcare, Madrid, Spain). The pH of the protein samples was checked and adjusted to an optimum pH of 8.8 and the protein concentration was later assessed by a RC-DC™ kit (Biorad, Madrid, Spain) following the manufacturer’s recommended protocol.

**Differential in gel electrophoresis (DIGE)**. Proteins were labelled using the CyDye DIGE Fluors minimal dyes for Ettan DIGE (GE Healthcare, Uppsala, Sweden), according to the manufacturer’s instructions for DIGE, prior to the isoelectric focussing (IEF). Briefly, 400 pmol of each dye (Cy3 or Cy5) was added to those samples containing 50 μg of protein, which were then incubated for 30 min on ice in the dark. The labelling reaction was quenched by adding 1 μL of L-lysine (10 mM) and incubated on ice for 10 min. A sample pool, with the same amount of protein from each sample was used as the internal reference to permit the normalization and was labelled with Cy2 (Table 1).

**Isoelectric focusing (IEF).** From the previous 2D gels, the proteome appeared to be very acidic. Various approaches were used to improve the resolution. The best conditions were IPG strips with a pH between 4 and 7; the concentration of IPG buffer increased to 3%, instead of the 0.5–2% recommended by the manufacturer, and a combined passive and active rehydration. The labelled samples were mixed thoroughly, in a 1:1:1 ratio, as shown in Table 1. Finally, the mixed protein samples were diluted to 450 μL with a rehydration buffer along with 20 mM DTT and 3% IPG buffer pH 4–7 (GE Healthcare), and a trace amount of bromophenol blue. Samples were loaded on 24 cm IPG strips pH 4–7 (Immobiline DryStrip, GE Healthcare, Madrid, Spain) and IEF was carried out at 25 °C and protected from direct light. Strips were first passively rehydrated at 0 V for 7 h and then actively rehydrated at 50 V for 14 h, which accumulated 288 V·h. IEF was performed using an IPGphor 3 instrument (GE Healthcare) in the following stages: an initial step of 100 V for 5 h, followed by four step gradients of 500 V for 30 min, 500 V for 7 h, 1000 V until 800 V·h and 8000 V until 13,500 V·h. Finally, there was an 8000 V step until 45,000 V·h was reached. A total of 64,076 V·h was accumulated at the end.

The control and test samples were referred to with a B to indicate that it was a bacterial consortium, followed by the compounds used for the propagation for growth (G for glucose; E for ETBE) and the concentration used in mg/L. The roman numeral indicates the replicates used (*n* = 2). The internal standard is the pool of all samples, including the replicates. The labelled samples were mixed thoroughly, in a 1:1:1 ratio with the final protein concentration of 150 µg per gel.

**Second dimensional electrophoresis.** Following IEF, the IPG strips were equilibrated for 15 min in 5 mL of reducing equilibration buffer (6 M urea, 30% glycerol, 50 mM Tris HCl pH 8.8, 2% SDS, 10 mg/mL of DTT and traces of bromophenol blue), followed by 15 min incubation in 5 mL of an alkylating equilibration buffer (6 M urea, 30% glycerol, 50 mM Tris HCl pH 8.8, 2% SDS, 25 mg/mL of iodoacetamide and traces of bromophenol blue). The second dimension SDS-PAGE was performed on an Ettan™ DALTsix Electrophoresis System (GE Healthcare, Madrid, Spain) at 1 W/gel at 25 °C, overnight until the bromophenol blue reached the bottom of the gel. The entire electrophoresis unit was protected from direct light during the run.

**Image acquisition and image analysis.** DIGE gels were immediately scanned after the SDS-PAGE at their respective excitation/emission wavelengths of 488/520 nm for Cy2, 532/580 nm for Cy3 and 633/670 nm for Cy5 using Molecular Imager PharosFX™ Plus (Biorad, Madrid, Spain). The images were analysed with Progenesis Same Spots (version 3.3, nonlinear Dynamics Ltd., Newcastle upon Tyne, UK). The protein spots were detected and normalised with their volume ratio to the internal standard in their respective gel. The spots were then filtered with ANOVA (*p* ≤ 0.05) and a fold difference of 1.5, between the control and the conditions was chosen as the criterion for identifying the differentially expressed protein candidates. The hierarchical clustering and the principle component analysis (PCA) were performed to assess the overall changes in the responsive proteins in all conditions using Progenesis Same Spots.

**Spot Picking and In-gel digestion**. For spot picking, one preparative gel with 200 µg of protein lysate was run with the same electrophoretic parameters for the first and second dimension as DIGE gels. The preparative gel was stained overnight with Coomassie brilliant blue. Selected spots were excised using EXQuest™ Spot cutter (Biorad, Madrid, Spain) and manually digested with trypsin (sequencing grade; Promega, Madison, WI) overnight at 37 °C, according to the manufacturer’s instructions. The resulting tryptic peptides were concentrated, purified and desalted using ZipTip^®^ (Merck Millipore, Madrid, Spain).

**Mass spectrometry analysis**. Tryptic digests of each spot (1 μL) were loaded on the AnchorChip target plate (Brucker-Daltonics, Billerica, MA, USA) with α-cyano-4-hydroxycinnamic acid (CHCA) matrix. Both MS and MS/MS spectra were generated in the reflectron mode using an UltrafleXtreme MALDI-TOF/TOF mass spectrometer (Bruker-Daltonics, Billerica, MA, USA). MS/MS data were acquired with an average of 1000–2000 laser shots for each spectrum. The combined peptide mass fingerprint and MS/MS were analysed by ProteinScape v3.0 software (Bruker-Daltonics, Billerica, MA, USA) and searched against the NCBI and TrEMBL databases (with taxonomic restriction for bacteria) using the MASCOT 2.0 software (http://www.matrixscience.com). The parameters used for the search engine were: fragment mass accuracy of ±0.5 Da, peptide mass accuracy of 50 ppm, one tryptic missed cleavage, fixed modification for carbamidomethylation of cysteine and variable modification for the partial oxidation of methionine. The peaks were also filtered for known autocatalytic trypsin peaks. Only significant search results (*p* ≤ 0.05) were accepted but they were always verified manually with a MASCOT analysis.

## 3. Results and Discussion

The enrichment cultures were obtained by seeding the samples collected from the groundwater contaminated with ETBE, the initial concentration of which was 720 µg/L. The sample also consisted of MTBE, benzene, toluene, xylene and other hydrocarbons along with ETBE. The initial enrichment culture EB1 obtained by inoculating the initial gasoline-contaminated water grew readily with ETBE (dissolved in methanol) as a carbon source and reached maximum growth on day 7 (OD_600_ = 0.6). The bacterial growth was only 0.3 OD on day 7 with 50 mg/L of ETBE in EB3. This may be because of the absence of other chemicals (found in the initial gasoline-contaminated water sample) which could stimulate the growth of bacteria. However, methanol was withdrawn during EB4 and the bacterial consortium was forced to use ETBE as the sole carbon source in the subsequent subcultures.

### 3.1. Biodegradation Using Consortium B

**Figure 1:**
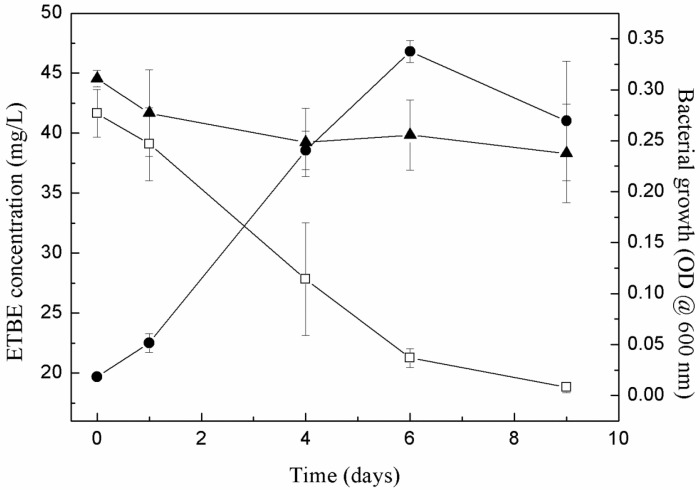
**Degradation of ETBE by bacterial consortium B (EB3).** The graph represents the degradation assay carried out with consortium B (EB3) with 50 mg/L of ETBE as the initial concentration. 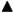 Control; 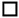 B consortium-EB3; 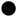 bacterial growth (OD at 600 nm). The error bars represent the standard deviation between the replicates (*n* = 2).

**Figure 2:**
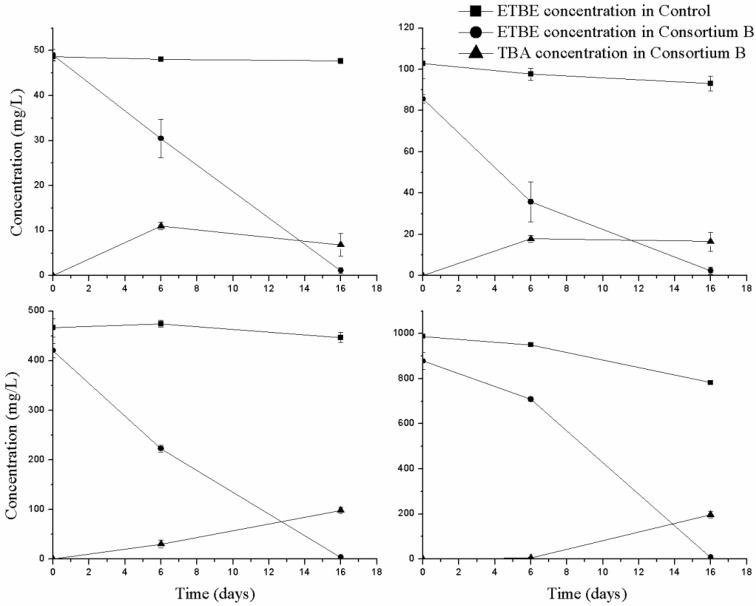
ETBE degradation and TBA formation in the degradation assay with different concentrations of ETBE by bacterial consortium B (EB11). The error bars represent the standard deviation between the replicates (*n* = 2). The graphs represent the degradation assay carried out with consortium B (EB11) with 50, 100, 500, and 1000 mg/L of ETBE as initial concentration.

The capacity of the enriched bacterial consortium to degrade ETBE was tested at two different stages of the enrichment phase (EB3 and EB11) to monitor for any changes in the degradation of ETBE over the subcultures. The biodegradation of ETBE by EB3 is shown in Figure 1. It can be observed that as the biomass increases, the biodegradation also increases. ETBE was degraded by about 50% in 6 days. Moreover, EB11 degraded 36% of the added 50 mg/L ETBE on day 6, which was lower than the previous experiment with EB3. This suggests that the growth factors or chemicals that support bacterial growth (including methanol), which were present more at the beginning, were lost in the subsequent subcultures. This was reflected in the growth: for example, the OD of EB11 was only 0.1 after 6 days whereas the OD of EB3 with 50 mg/L of ETBE was 0.3. The growth of the bacterial consortium EB11 increased to 0.3 OD but only after 16 days of incubation. Nevertheless, EB11 degraded 50, 100, 500 and 1000 mg/L of ETBE by about 98% in 16 days (Figure 2). The ETBE concentration of 10,000 mg/L was found to be inhibitory, which impeded the growth of the consortium, as measured on the 16th day (data not shown). The degradation rate for 50 mg/L of initial ETBE was 3 mg/L·h. For the other concentrations at 6 and between 7 and 16 h, the degradation rates were, respectively, 8.3 and 3.3 mg/L·h for 100 mg/L initial ETBE; 32.9 and 21.9 mg ETBE/L·h for 500 mg/L initial ETBE and finally 28.2 and 70.2 mg/L·h for 1000 mg/L. The ETBE degradation rate of consortium B is higher than the reported consortium Pz1-ETBE and MC-IFP (consisting of *R. wratislaviensis* IFP 2016, *R. aetherivorans* IFP 2017 and *A. tertiaricarbonis* IFP 2003) the degradation rates of which were 0.91 mg/L·h and 0.83 mg/L·h [10].

As a result of the initial ETBE biodegradation by consortium B, we used GC-MS to identify TBA, one of the primary products described in ETBE metabolism, in both EB3 and EB11 degradation samples (Supplementary Figure S1). The concentration of TBA was quantified using GC-FID for 6 and 16 days, in parallel with the ETBE biodegradation studies with EB11 (Figure 2). No significant loss of ETBE was detected in the abiotic controls, which discounts the possibility of other modes of the ETBE degradation. Similarly, TBA was not generated in the abiotic controls. The TBA accumulation can be correlated to the partial degradation of ETBE. The results were similar when the ETBE and MTBE degradation by a mixture of bacterial strains led to recalcitrant TBA [8]. Following the same time period TBA levels accumulated, no matter what concentrations of ETBE were used. We also inferred that about 20% of the initial ETBE concentration was converted to TBA after 16 days (Figure 2). In order to have a clearer idea about the complete mineralization of TBA, it should be monitored for longer periods of time. The ability of consortium B (EB11) to degrade 50 mg/L of MTBE, TBA, and BTX during its growth was tested independently. It was found that both MTBE and TBA did not support the growth of the bacterial consortium but, in the case of TBA, a less than 10% degradation was observed on day 9 after which the concentration remained constant. The initial concentration of the compounds used here was 50 mg/L. Higher concentrations (100 mg/L and 500 mg/L) of TBA and MTBE were also tested but no degradation was observed. It should also be noted that when TBA was tested without ETBE, the TBA degradation was very slow or negligible. This suggests that the TBA degradation may need ETBE as a growth substrate. None of the BTX compounds supported either growth or degradation of the ETBE by EB11, in contrast to what we previously found with consortium A [19]. Hence, it is clear that the additives are required if consortium growth is to improve degradation [27]. We attempted to detect HIBA in the biodegradation samples, but were unable to do so with our methods. We inferred that EB11 adapted to ETBE by inducing the molecular pathways to metabolize it. However, the biomass of EB11 decreased in presence of ETBE. This may be due to TBA being toxic to the consortium at the levels accumulated in the medium. Further, additives can be tested to improve the biomass of the consortium without losing the degradation ability of EB11 for subsequent large-scale biodegradation studies. Nevertheless, a bacterial consortium capable of partially degrading higher concentrations of ETBE was enriched. 

**Bacterial identification:** Following four days of incubation, fourteen colonies appeared on the ETBE plates with EB2. The colonies appeared on the washed agar plates with 50 mg/L ETBE from which any other traces of soluble carbon had been removed [26]. Nine of the fourteen bacterial colonies were successfully identified by the 16S rDNA identification. All of the bacteria identified were aerobic and rod shaped, and belonged to the β-proteobacteria, γ-proteobacteria and actinobacteria class of bacteria. The colonies belonged to five bacterial genera (*Xanthomonas*, *Herbaspirillum*, *Pseudacidovorax*, *Arthrobacter* and *Pseudomonas*) and their respective sequences were submitted to GenBank (Table 2). In particular, the genus *Pseudomonas* is very well known for degrading many xenobiotic compounds and harbouring the genes involved in the degradation process. Several members of this genus have been reported to be involved in the degradation of hydrocarbons [28,29], aromatic compounds [30], pyrenes [31], and petroleum tar [32]. *Xanthomonas* is known to be able to degrade petroleum hydrocarbons [33], toluene and xylene isomers [34], hexachlorocyclohexane [35], and herbicides [36]. *Arthrobacter* has been reported to degrade MTBE [37] and *Herbaspirillum* to degrade chlorophenol [38], fluoranthene [39] and anthracene [40]. Moreover, only two single colonies were found on the ETBE plates plated with EB11. The colonies were identified using the 16S rDNA identification and the sequences matched *Xanthomanas* and *Pseudomonas* sp. (Table 2). We concluded that *Xanthomonas* and *Pseudomonas* sp. were conserved and selected with the stress condition from the EB2 to EB11 subcultures. These two bacterial genera have been reported to participate in bioremediation. We continued our degradation studies with the selected bacterial consortium and not with the single bacterial strains obtained, in an attempt not to lose other unidentified bacterial strains in the community and to maintain their possible synergistic effects on the degradation. In support of this, a consortium consisting of *Pseudomonas* and *Rhodococcus koreensis* was able to grow on MTBE and TBA [27]. However, its capacity to degrade MTBE and TBA was low and additional substrates (2-propanol) were required when individual isolates were tested. Other consortia were identified from the polluted samples, which were dominated by *Mesorhizobium* and *Hydrogenophaga* [41]; and Comamonadaceae and Gammaproteobacteria families [42,43] as ETBE biodegraders.

### 3.2. Comparative Metaproteomic Analysis of the ETBE-Degrading Consortium

**Figure 3:**
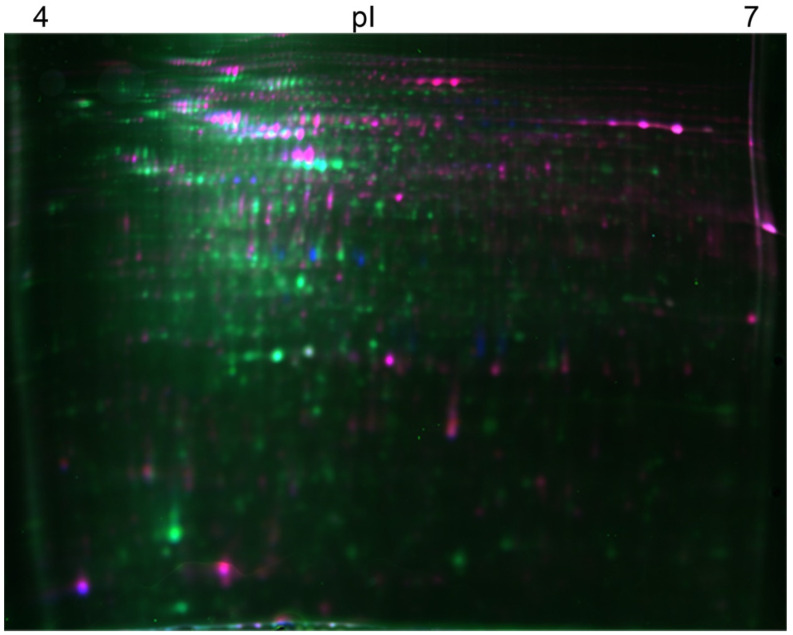
**2D-DIGE gel showing the electrophoretic map of the soluble proteins in bacterial consortium B.** The image shows a representative picture of three independent gels. p*I*: Isoelectric point. Soluble proteins from bacterial consortium B grown in 0.5 g/L of glucose were labelled with Cy3 (green) and proteins from bacterial consortium B grown in 500 mg/L of ETBE were labelled with Cy5 (purple). The pooled internal standard was labelled with Cy2 (blue).

A metaproteomic analysis was carried out using the 2D-DIGE gel approach to gain an insight into the mechanism underlying the biodegradation of ETBE, TBA accumulation and the general cellular response of the bacterial consortium B (EB11). The gel provides a map of intact proteins, which reflects changes in the protein expression level, isoforms and post-translational modifications. It was essential to make a comparative study with a control in order to retrospect how the bacterial consortium differs from a less stressful environment. However, it is impossible to mimic a natural environment in which microbial communities use various substrates with alternating physiological conditions [23]. Hence, choosing a control for a complex bacterial consortium is extremely challenging. Here in this study, we use glucose as a control for the following reasons: it is a simple carbohydrate monomer which is bioavailable, bacterial cultures can readily grow on it, and it only stimulates a low number of proteins involved in the catabolism and cell growth [44]. Nevertheless, the control was selected bearing in mind that the cells are in the stationary phase in both cases. Thus, the bacterial cells grown in ETBE for 16 days (when the extent of the ETBE degradation was at its highest, above 95%) were examined with a control, propagated in glucose. A total of 1789 protein spots were detected (Table 1, Figure 3). A one-way ANOVA analysis (*p* ≤ 0.05) revealed that 241 spots were differentially expressed with a minimum ratio of a 1.5-fold change in the samples with ETBE. These protein spots were examined for definite patterns by realignment and noise levels, and 92 spots were selected for analysis. Of these, 77 were more abundant in consortium B treated with ETBE at both concentrations of 500 and 1000 mg/L, than in the control. The differential protein data sets were also subjected to PCA to investigate the inter- and intra-group relationships among the given conditions and to identify the protein groups responsible for the correlated variations. There is a clear difference between the test sample (bacterial consortium with ETBE) and the control (bacterial consortium with glucose), which are grouped together. The replicates (in different gels) clustered closely, indicating that the biological variation is responsible for the separation of the different treatments (Supplementary Figure S2), which explains the quality of the data presented here. The power analysis made using the software Progenesis, revealed that the use of duplicates is sufficient to obtain a more than 80% confidence in the statistical analysis (Supplementary Figure S3). As expected, we were able to obtain three different clusters from the protein datasets, based on similar protein abundance profiles (Supplementary Figures S4 and S5). The first cluster revealed the less abundant proteins in the ETBE-induced conditions. The other two clusters showed proteins that are more abundant in the presence of ETBE (the abundance of each protein depended on the concentration of ETBE used). Thus, it is clear that the two different concentrations of ETBE used here had an effect on the protein profile of the bacterial consortium.

#### Identification of the Protein Spots

**Figure 4:**
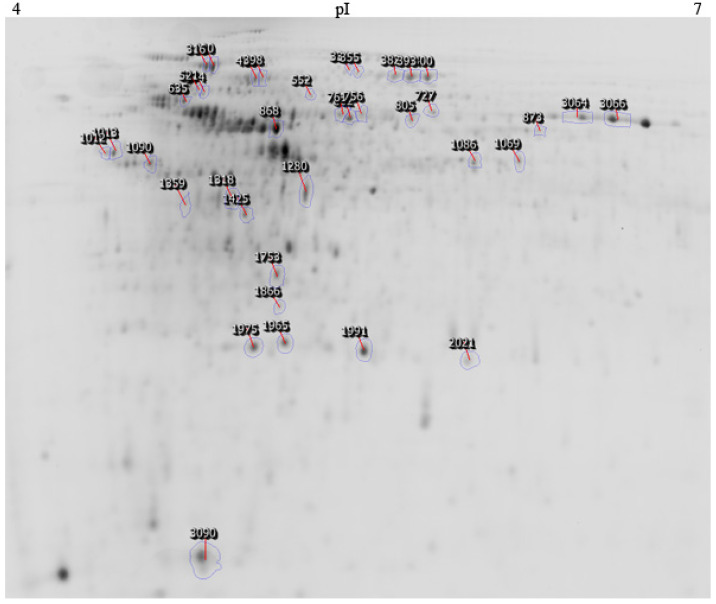
**2D gel showing the selected protein spots.** The image shows a preparative gel stained with Coomassie brilliant blue representing the 38 protein spots selected for analysis in MALDI-TOF/TOF. pI: Isoelectric point.

**Figure 5:**
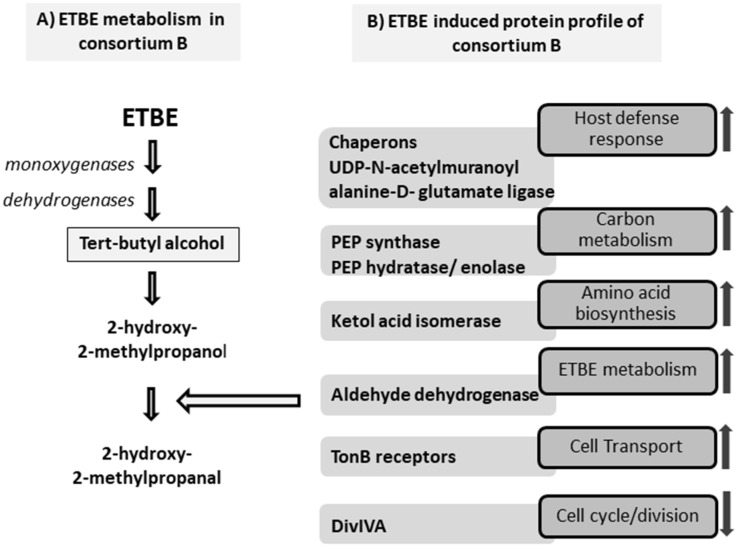
**Schematic interpretation of the global response of bacterial consortium B in the presence of ETBE.** Panel (**A**) represents the possible route of the ETBE metabolism followed in consortium B. Tert-butyl alcohol was identified by a GC-MS analysis, which is represented in the box. Panel (**B**) represents the proteins that are induced in bacterial consortium B in response to ETBE. The abundance of the protein is indicated in boxes with arrows pointing up or down to represent the more or less abundant proteins in the ETBE-induced state. Aldehyde dehydrogenase is identified in the proteomic analysis. It is believed to be involved in the ETBE metabolism and is abundant, as indicated by the arrow.

Finally, of all of the statistically different expressed spots, the thirty-eight most abundant were analysed by MALDI-TOF/TOF (Figure 4). However, the complexity of the environmental metaproteomic data meant that only 12 proteins were successfully identified (Table 3, Figure 5). It should be taken into account that the data on the environmental bacteria are incomplete in the proteomic databases, which makes the analysis difficult [45]. Of the 12 spots, five (316, 552, 727, 1013, 1280) increased with increasing concentrations of ETBE (Supplementary Figure S5).

**Proteins associated with the ETBE metabolism.** Two aldehyde dehydrogenases were identified at different spot locations in the gel (spots 410 and 552). Of these, one (spot 552) was abundant at both concentrations of ETBE with a 4.2-fold change. It corresponded to the NAD-dependent aldehyde dehydrogenase and s-ethyl dipropylthiocarbamate (ETPC) inducible aldehyde dehydrogenase in th NCBI and TrEMBL database searches. Aldehyde dehydrogenase is reported to be involved in the lower pathway of the MTBE degradation in *Mycobacterium austroafricanum* IFP 2012 [46] and is encoded by the gene *mpd*C. This enzyme catalyzes the dehydrogenation of hydroxyisobutyraldehyde and converts it into HIBA. The protein identified matched *Rhodococcus erythropolis* PR4 and *Gordonia bronchialis* or *Rhodococcus bronchialis* as the nearest hits. The identification of this protein suggests that the consortium consists of bacterial species that can metabolize ETBE and its metabolites. However, another aldehyde dehydrogenase identified (spot 410; 1.6× fold) was less abundant in BE500 and BE1000, than in the control. The possibility of isoforms being present in the protein or modifications while processing can be ruled out as the induction levels (fold change) of the two proteins are completely different, which implies that the two spots belong to two different proteins. However, not all dehydrogenases are responsible for ETBE degradation [47]. According to Hristova et al. [47], the hydroxyisobutyraldehyde (HIBAL) dehydrogenase was difficult to identify as there are 11 genes belonging to the aldehyde dehydrogenase superfamily in the *M. petroleiphilum* PM1 genome. However, only one was predicted to be the right candidate, which showed an identity of 33% to MpdC of *M. austroafricanum* IFP 2012 with a significant 1.4-fold abundance. Therefore, we can predict that the aldehyde dehydrogenase corresponding to protein spot 552 is the most likely candidate to be involved in the ETBE degradation. It matches the theoretical MW (55) and pI (4.9) perfectly. However, other enzymes involved in the initial cleavage of ETBE cannot be identified by protein-spot selection, based on the most abundant fold change. TBA was identified as an intermediate metabolite (Supplementary Figure S1) and the identification of the probable aldehyde suggests that our ETBE-degrading consortium probably follows the pathway proposed in Figure 5 (adapted from Rohwerder et al. [48]).

**Proteins related to carbon metabolism and biosynthetic pathways.** Phosphoenolpyruvate (PEP) synthase (spot 316), which increases 5 fold in consortium B, is involved in such biological processes as the gluconeogenesis pathway, the pyruvate metabolism and phosphorylation reactions. It is a key enzyme in carbon fixation because it provides substrates for multiple biosynthetic pathways. PEP synthase was reportedly abundant (5 fold) in *Alkanivorax* sp. in the presence of alkanes [49]. Phosphopyruvate hydratase (spot 1013, 2.5 fold) belongs to the lyase superfamily of bacterial enzymes. This enzyme is mainly involved in the synthesis of phosphoenolpyruvate as predicted by the KEGG database. Among other functions, it takes part in glycolysis and gluconeogenesis, the biosynthesis of secondary metabolites, methane metabolism, and other microbial metabolisms in different environments. Similarly, the protein spot 1086, which showed an increase of 7.2 fold with ETBE, was identified as enolase, an ortholog of the protein phosphopyruvate hydratase and with similar predicted biological functions. Glyceraldehyde-3-phosphate dehydrogenase (spot 1280, 6.5 fold) also participates in carbon central metabolism [50].

**Chaperones induced by ETBE**. Chaperone DnaK (spot 727) increased 4.8 fold in cells grown in ETBE, compared to the control with glucose. Chaperones are reported to be involved in numerous molecular functions, including nascent protein folding and the assembly for protein activation, protein refolding, translocation across membranes, heat-shock responses (housekeeping function), and defence against stress [51]. The increase of this protein can reflect either an increased demand for the folding of newly synthesized proteins or a stress response of the bacterial cell to the given environment. It is evident that the carbon flow increases along the biosynthetic pathways and that, naturally, chaperones are required to process the synthesized molecules. The molecular chaperones DnaK and GroEL are stimulated in the MTBE-grown *M. austroafricanum* IFP 2012 [46] and chaperone activity was found in *A. xylosoxidans* MCM1/1, in the presence of MTBE [52], which supports our findings.

The metaproteomic analysis gave an insight into the ETBE-degrading consortium where the metabolism is concentrated in the ETBE metabolism, carbon metabolism and cell survival. A proteomic tool was also used to assess the MTBE biodegradation in groundwater [53]. Even though proteins of MTBE degrading microorganisms were present, the MTBE degradative proteins were only found in one of the two stations analysed.

Although the proteomic analysis was robust and significantly found large differences between the two conditions in our study (glucose and ETBE), identifying the proteins was a great challenge as very little data significantly matched an identified protein from the complex environmental bacterial consortium. Finally, increasing the availability of the genome data from the environmental samples and improving the de novo sequencing strategies will improve the analysis and make proteomes easier to identify, thereby facilitating a better understanding of the adaptations of bacterial communities [54].

## 4. Conclusions

A bacterial consortium capable of degrading ETBE was enriched and isolated from a gasoline-contaminated water body. The isolated bacterial consortium was able to grow on ETBE as the sole carbon and energy source. This consortium, which consisted of *Pseudomonas* sp. and *Xanthomonas* sp. was found to adapt and use considerable concentrations of ETBE, producing TBA as a metabolite. The metaproteomic profile gave an insight into the metabolic adaptations of bacterial consortium B in response to ETBE at higher concentrations. The response of the bacterial consortium to ETBE was discussed and the enzymes identified in the proteomic analysis partly mirrored the metabolism of ETBE. Thus, the presence of ETBE modified the abundance of proteins associated with the ETBE metabolism, carbon metabolism, and chaperones. The present study demonstrates the potential of an isolated bacterial consortium to partially degrade higher concentrations of ETBE, which can be useful in the bioremediation of sites heavily contaminated with ETBE. Moreover, the metaproteomics of an ETBE-degrading bacterial consortium has been studied for the first time.

## Figures and Tables

**Table 1:** DIGE labelling for the different samples.

Gel Number	CyDye	Sample (50 µg)
1	Cy2	Internal standard
	Cy3	BGI
	Cy5	BE500II
2	Cy2	Internal standard
	Cy3	BE1000I
	Cy5	BGII
3	Cy2	Internal standard
	Cy3	BE500I
	Cy5	BE1000II

**Table 2:** Identification of the bacterial strains from the enriched bacterial consortium B by 16S rDNA.

Colony Name	GenBank Accession Number	Strain (GenBank) ^a^	Strain (RDP) ^b^	Class
EB2-1	KU898255	*Uncultured Xanthomonas* sp.	*Xanthomonas* sp.	γ-proteobacteria
EB2-2	KU898256	*Herbaspirillum* sp. MMD15	*Herbaspirillum rubrisubalbicans*	β-proteobacteria
EB2-4	KU898257	*Pseudacidovorax* sp. ptl-2	*Pseudacidovorax intermedius*	β-proteobacteria
EB2-7	KU898258	*Arthrobacter* sp. 2073	*Arthrobacter nicotinovorans*	Actinobacteria
EB2-10	KU898259	*Pseudacidovorax intermedius*	*Pseudacidovorax intermedius*	β-proteobacteria
EB2-11	KU898260	*Pseudacidovorax intermedius*	*Pseudacidovorax intermedius*	β-proteobacteria
EB2-14	KU898261	*Pseudomonas* sp.	*Pseudomonas veronii*	γ-proteobacteria
EB11-1	KU898262	*Pseudomonas fluorescens* hswX151	*Pseudomonas fluorescens*	γ-proteobacteria
EB11-2	KU898263	*Xanthomonas* sp. FSBSY16	*Xanthomonas* sp. *CU12*	γ-proteobacteria

EB2 and EB11 denote the respective consortium B enrichment culture from which the colonies were isolated, followed by the colony number. ^a^ As obtained from the sequence match to GenBank Blast. ^b^ As obtained from the sequence match to the RDP 10 database.

**Table 3:** Protein identification using MALDI-TOF/TOF-MS.

SPOT ID	Accession Number	Protein identified (Enzyme Code)	MW [kDa]	pI	Species Homology	Fold Change	Upregulation	Biological Function
316	gi|21231605	Phosphoenolpyruvate synthase ^a^ (EC 2.7.9.2)	86.2	5	*Xanthomonas campestris*	5.12	ETBE 500 ETBE 1000 *	Gluconeogenesis, Carbohydrate biosynthesis
	B0RSD3_ XANCB	Phosphoenolpyruvate synthase ^b^ (EC 2.7.9.2)	86.2	5	*Xanthomonas campestris*			
	gi|77165106	Fumarate hydratase ^a^ (EC 4.2.1.2)	50.6	7.1	*Nitrosococcus oceani*			Carbohydrate metabolism, TCA cycle
320	gi|84494866	Nicotine dehydrogenase chain C ^a^ (EC 1.5.99.4)	88.7	4.7	*Janibacter* sp.	4	ETBE 500 ETBE 1000	Oxidoreductase activity
	A3THC9_ 9MICO	Nicotine dehydrogenase chain C ^b^ (EC 1.5.99.4)	88.7	4.7	*Janibacter* sp.			
393	H0BRS3_ 9BURK	Aldehyde oxidase (EC 1.2.3.1) and xanthine dehydrogenase molybdopterin-binding protein ^b^ (EC 1.17.1.4)	85.1	5.5	*Acidovorax* sp. NO-1	10.70	ETBE 500 ETBE 1000	Oxidoreductase activity Purine metabolism
400	gi|84625297	Putative TonB-dependent receptor ^a^ (EC 3.6.3.34)	84.5	5.4	*Xanthomonas oryzae pv. oryzae*	14.89	ETBE 500 ETBE 1000	Receptor activity, transporter activity
410	gi|77166367	Aldehyde dehydrogenase ^a^ (EC 1.2.1.16)	56.9	5.2	*Nitrosococcus oceani*	1.60	Glucose	Oxidoreductase activity Glycolysis/Gluconeogenesis Microbial metabolism in diverse environments
552	C0ZVY3_ RHOE4	Aldehyde dehydrogenase ^b^ (EC 1.2.1-)	55.1	4.8	*Rhodococcus erythropolis*	4.20	ETBE 500 ETBE 1000 *	
	D0LAD0_ GORB4	Aldehyde dehydrogenase (NAD(+)) ^b^ (EC 1.2.1.3)	55.3	4.9	*Gordonia bronchialis*			
	gi|1174662	EPTC-inducible aldehyde dehydrogenase ^a^ (EC 1.2.1.3)	55	4.9	-			
727	gi|152968176	Chaperone protein DnaK ^a^	66.4	4.7	*Kineococcss radiotolerans* SRS30216	4.80	ETBE 500 ETBE 1000 *	Protein folding, Stress response
	K1ET82_ 9MICO	Chaperone protein DnaK ^b^	67.1	4.4	*Janibacter hoylei* PVAS-1			
	gi|84497587	molecular chaperone DnaK ^a^	67.9	4.5	*Janibacter* sp. HTCC2649			
1013	gi|84497843	Phosphopyruvate hydratase ^a^ (EC 4.2.1.11)	45.9	4.4	*Janibacter* sp. HTCC2649	2.50	ETBE 500 ETBE 1000 *	Glycolysis/Gluconeogenesis
	gi|21221535	Phosphopyruvate hydratase ^a^ (EC 4.2.1.11)	45.5	4.3	*Streptomyces coelicolor* A3			
	H0QHC6_ ARTGO	Enolase ^b^ (EC:4.2.1.11)	45	4.3	*Arthrobacter globiformis*			
1086	I0KWX5_ 9ACTO	Enolase ^b^ (EC 4.2.1.11)	45	4.5	*Micromonospora lupini* str. Lupac 08	7.20	ETBE 500 ETBE 1000	Glycolysis/Gluconeogenesis
	C0FPT6_ 9FIRM	Enolase ^b^ (EC:4.2.1.11)	50.3	4.8	*Roseburia inulinivorans* DSM 16841			
1280	gi|121998872	UDP-N-acetylmuramoylalanine--D-glutamate ligase ^a^ (EC 6.3.2.9)	46.1	5.1	*Halorhodospira halophila* SL1	6.50	ETBE 500 ETBE 1000 *	Cell wall biogenesis/degradation
	D0WEA8_ 9ACTN	Glyceraldehyde-3-phosphate dehydrogenase, type I ^b^ (EC 1.2.1.12)	35.8	5.2	*Slackia exigua*			Glycolysis
1425	H0QLW5_ ARTGO	Ketol-acid reductoisomerase ^b^ (EC 1.1.1.86)	37.2	4.7	*Arthrobacter globiformis*	2.45	ETBE 500 ETBE 1000	Branched-chain amino acid biosynthesis
	E3BB64_ 9MICO	Ketol-acid reductoisomerase ^b^ (EC 1.1.1.86)	37.2	4.7	*Dermacoccus* sp. Ellin185			
1753	A0JVA2_ ARTS2	DivIVA family protein ^b^	25	5	*Arthrobacter* sp. (strain FB24)	18.50	Glucose	Cell cycle Cell division

Proteins were identified by MS/MS spectra using the Mascot search engine. The number of identifications for each protein spot are the number of hits obtained from the respective database. The biological function of the proteins was identified using the UniProtKB database. MS/MS scores vary from 17.8 to 168, with an average of 57. ^a,b^ identified using the NCBI and TREMBL databases, respectively. * Highest protein abundance in the ETBE concentration used. Concentration expressed in mg/L.

## Supplementary Materials

The following supporting information can be downloaded at: https://www.mdpi.com/article/10.3390/microorganisms10122331/s1, Figure S1: GC-MS spectra of the identified tert-butyl alcohol; Figure S2: Principal component analyses of all the samples; Figure S3: Power analysis generated by the Progenesis Same Spot software; Figure S4: Hierarchical clustering patterns of protein spots; Figure S5: Expression patterns of all the identified protein spots.
