# Supplementary figures and images for: Comparative Proteomic Analysis of an Ethyl Tert-Butyl Ether-Degrading Bacterial Consortium
Source: Microorganisms. 2022 Nov 25;10(12):2331. doi: 10.3390/microorganisms10122331 (PMC9781318; doi:10.3390/microorganisms10122331)

## Supplementary Figures

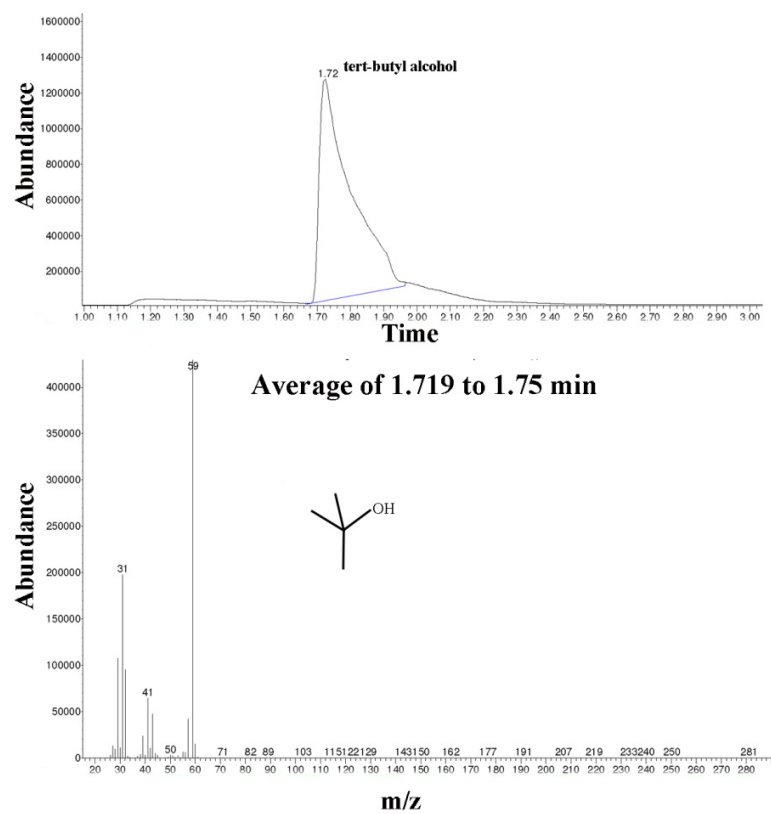

Figure S1

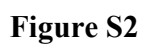

### Figure S2

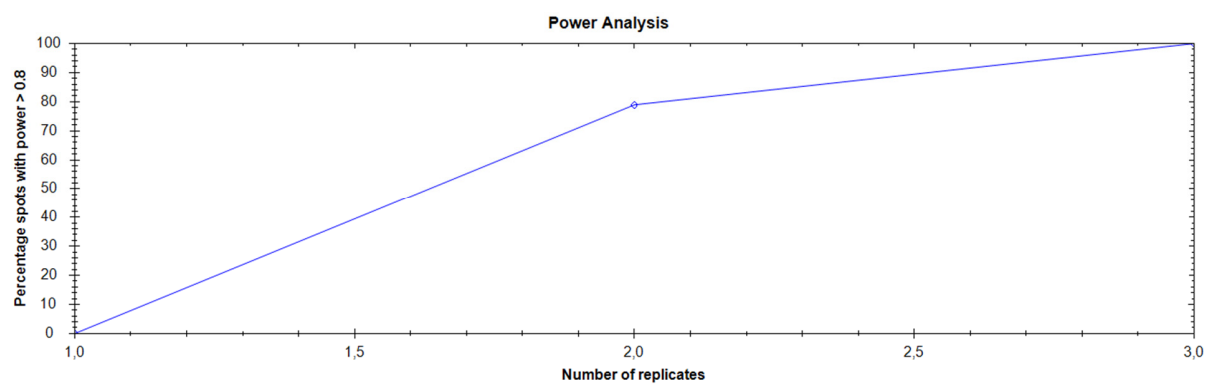

**Figure S3**

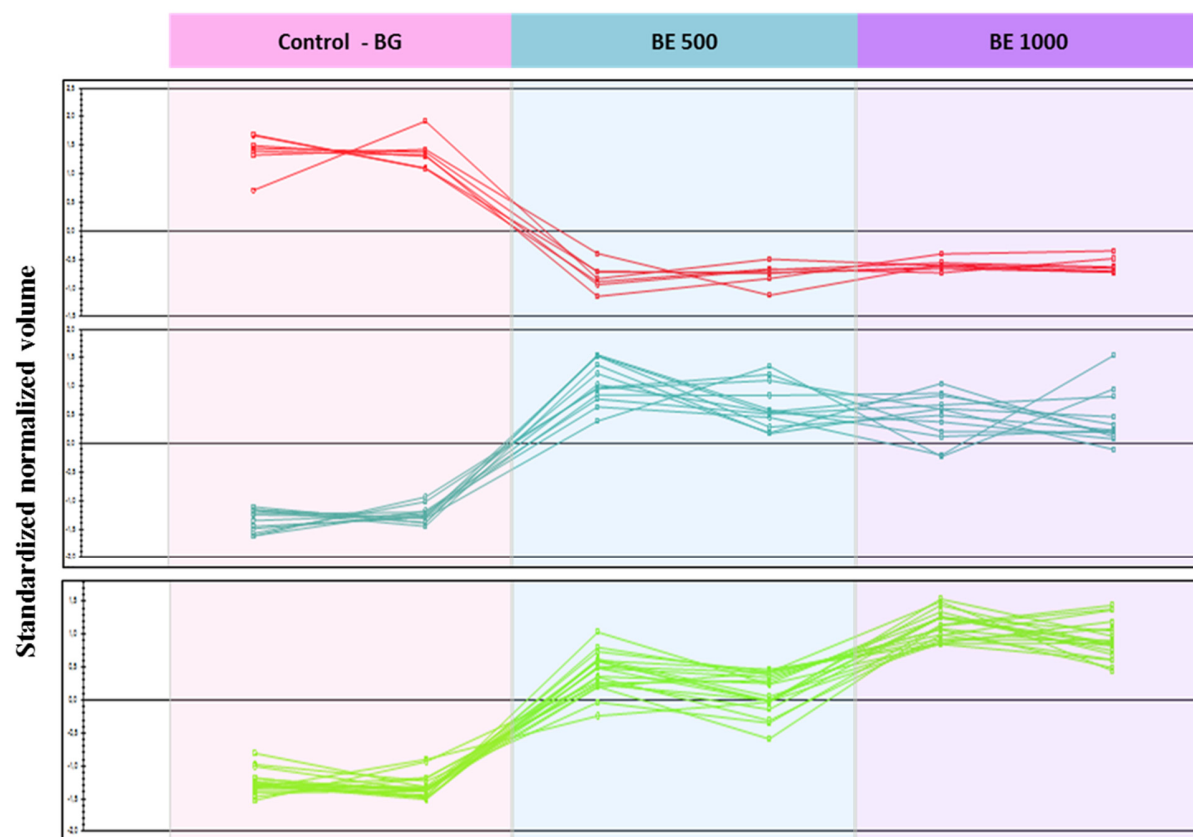

Figure S4

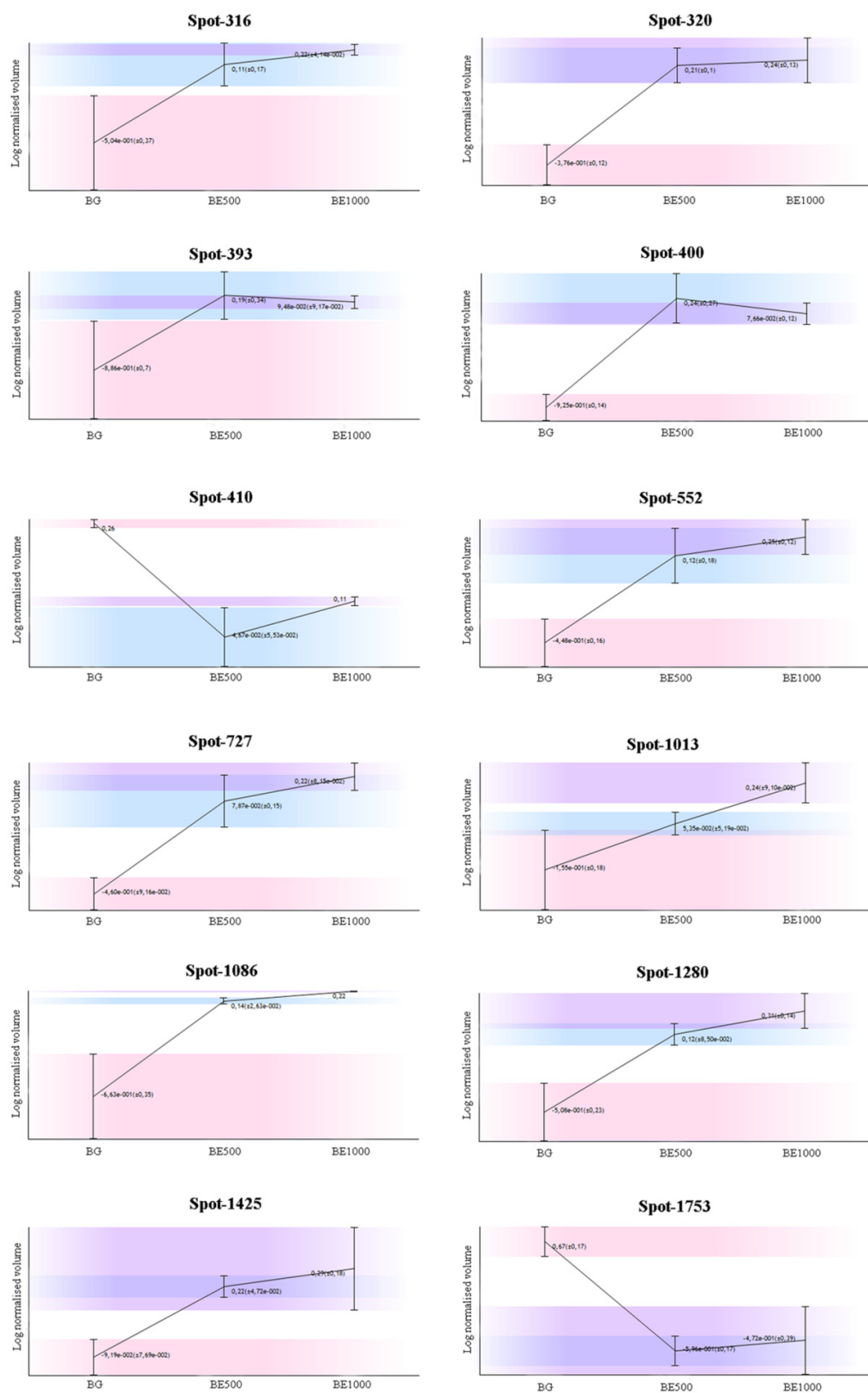

**Figure S5**

Supplement: Supplementary file 1 [file microorganisms-10-02331-s001.zip › microorganisms-2022517-supplementary.pdf]
